# Hemodynamic Heterogeneity in Community-Acquired Sepsis at Intermediate Care Admission: A Prospective Pilot Study Using Impedance Cardiography

**DOI:** 10.3390/healthcare13212686

**Published:** 2025-10-23

**Authors:** Gianni Turcato, Arian Zaboli, Lucia Filippi, Fabrizio Lucente, Michael Maggi, Alessandro Cipriano, Massimo Marchetti, Daniela Milazzo, Christian J. Wiedermann, Lorenzo Ghiadoni

**Affiliations:** 1 Department of Internal Medicine, Intermediate Care Unit, Hospital Alto Vicentino (AULSS7), 36014 Santorso, Italy; gianni.turcato@yahoo.it (G.T.); lucia.filippi@aulss7.veneto.it (L.F.); fabrizio.lucente@aulss7.veneto.it (F.L.); michael.maggi@aulss7.veneto.it (M.M.); massimo.marchetti@aulss7.veneto.it (M.M.); daniela.milazzo@aulss7.veneto.it (D.M.); 2 Health Professions Management, South Tyrolean Health Authority (SABES-ASDAA), 39100 Bolzano, Italy; 3 Emergency Department, Nuovo Santa Chiara Hospital, Azienda Ospedaliero-Universitaria Pisana, 56100 Pisa, Italy; alessandrocipriano@gmail.com; 4 Institute of General Practice and Public Health, Province College for Health-Care Professions “Claudiana”, 39100 Bolzano, Italy; 5 Department of Clinical and Experimental Medicine, University of Pisa, 56100 Pisa, Italy; lorenzo.ghiadoni@unipi.it

**Keywords:** sepsis, hemodynamic monitoring, impedance cardiography, stroke volume, total peripheral resistance index, cardiac power index, fluid responsiveness, intermediate care unit, community-acquired infections

## Abstract

**Background**: Sepsis is a heterogeneous syndrome in which patients with similar clinical presentations at admission may exhibit markedly different treatment responses and outcomes, suggesting that comparable macroscopic features can conceal profoundly distinct perfusion and hemodynamic states. **Aim**: This study aimed to characterize the hemodynamic profile of patients with community-acquired sepsis, assess its correlation with macro-hemodynamic indices, compare fluid responders with non-responders, and explore the prognostic value of early identification of a feature consistent with distributive shock. **Methods**: A prospective observational pilot study was conducted in the Intermediate Medical Care Unit (IMCU) of Ospedale Alto Vicentino (Santorso, Italy), September 2024–May 2025. 115 consecutive adults with community-acquired sepsis underwent NICaS^®^ bioimpedance assessment at IMCU admission. Sepsis was diagnosed at IMCU admission as suspected/confirmed infection plus an acute increase in total Sequential Organ Failure Assessment (SOFA) ≥ 2 points. Hemodynamic indices were analyzed in relation to the Sequential Organ Failure Assessment (SOFA) score and mean arterial pressure (MAP), fluid responsiveness, and 30-day mortality. **Results**: Hemodynamics were heterogeneous across patients and within SOFA strata. SOFA showed no correlation with SV, SI, CO, or CI; weak inverse associations for TPR (r = −0.198, *p* = 0.034) and TPRI (r = −0.241, *p* = 0.009) were observed. MAP did not correlate with SV, SI, CO, or CI, but correlated positively with TPR (r = 0.461) and TPRI (r = 0.547) and with CPI (ρ = 0.550), all *p* < 0.001. A distributive profile was present in 21.7% (25/115), increasing with higher SOFA (*p* = 0.033); only 20% of those with this profile had MAP < 65 mmHg at admission. Fluid non-responders (27.8%) had lower resistance and higher CI (4.1 vs. 3.4 L/min/m^2^; *p* = 0.015). The distributive profile was not associated with 30-day mortality (log-rank *p* = 0.808). **Conclusions**: In IMCU patients with community-acquired sepsis, macro-indices (SOFA, MAP) correlate poorly with the underlying hemodynamic state. Early noninvasive profiling reveals within-SOFA circulatory heterogeneity and may support operational, individualized resuscitation strategies; these pilot findings are hypothesis-generating and warrant prospective interventional testing.

## 1. Introduction

Sepsis is a time-sensitive, clinically complex, and potentially lethal syndrome that remains a leading cause of mortality in industrialized countries, with persistently high hospitalization and case-fatality rates [1,2]. It is defined as infection associated with organ dysfunction, operationalized as a Sequential Organ Failure Assessment (SOFA) score ≥ 2 [3]. Yet, this classification provides only a coarse approximation of severity and does not capture the marked pathophysiological heterogeneity observed in practice, as illustrated by the wide gradients in mortality across SOFA strata [4,5,6]. Following the 2021 Surviving Sepsis Campaign, sepsis is conceptualized as life-threatening organ dysfunction due to infection [3]. Because the SOFA score summarizes multi-organ dysfunction and does not directly capture macro-hemodynamic status, we investigated whether noninvasive cardiovascular indices can reveal circulatory heterogeneity among patients with similar SOFA levels, thereby complementing SOFA in characterizing perfusion phenotypes.

Current guidelines stipulate that meeting sepsis criteria prompt immediate fluid resuscitation, which continues to represent the cornerstone of management [7,8]. This approach, historically grounded in the assumption that organ dysfunction primarily reflects tissue hypoperfusion and intravascular volume deficit, has been standardized across patients despite ongoing debate [9,10]. However, it presumes relative hemodynamic homogeneity, whereas clinical experience and recent evidence highlight substantial variability in circulatory profiles and often unpredictable responses to fluids, even within seemingly comparable patient groups [11,12].

Systemic inflammation in sepsis induces a cascade of vascular and perfusion abnormalities—endothelial dysfunction, increased permeability, and altered vasoregulation—that originate in the microcirculation and only later manifest as macrocirculatory derangements [13,14]. Consequently, macroscopic indices such as SOFA score, mean arterial pressure (MAP), or lactate, though widely used to guide therapy, may aggregate patients into broad prognostic categories while concealing profoundly different hemodynamic states. Despite their prognostic value, these parameters therefore provide only a low-resolution picture of the underlying circulatory complexity [15,16].

This gap raises important questions. Can early noninvasive hemodynamic assessment reveal clinically meaningful perfusion phenotypes in patients with community-acquired sepsis that remain invisible to conventional indices? Do such phenotypes correlate with fluid responsiveness or with macro-hemodynamic surrogates such as SOFA and MAP? And could their early identification support a more individualized approach to circulatory management? Addressing these questions is particularly relevant outside the intensive care unit, where most sepsis patients are initially managed and where noninvasive tools could be integrated into routine workflows.

In practice, early noninvasive profiling could help prioritize vasopressors over additional fluids when a high-output/low-resistance pattern is present, or conversely optimize preload when flow is depressed—decisions that align with guidance favoring dynamic, flow-based reassessment rather than static thresholds in the first hours of resuscitation [3]. Integrating such bedside information at IMCU admission may therefore provide the operational granularity needed to tailor initial hemodynamic support.

Against this background, the present pilot study investigated consecutive patients with community-acquired sepsis admitted to an intermediate care unit. The aim was to characterize their admission hemodynamic profiles using noninvasive impedance cardiography, evaluate correlations with SOFA and MAP, examine differences between fluid responders and non-responders, and explore the short-term prognostic implications of a profile consistent with distributive shock.

This pilot study was designed to explore whether early, noninvasive hemodynamic profiling can uncover clinically relevant circulatory heterogeneity in patients with community-acquired sepsis admitted to an intermediate care unit. Specifically, the objectives were to:

- Characterize the spectrum of hemodynamic profiles present at admission.
- Assess whether conventional macro-hemodynamic indices, namely SOFA score and MAP, and reliably reflect these profiles, with particular attention to the distributive-shock phenotype.
- Investigate the relationship between baseline hemodynamic profiles and the response to a standardized fluid challenge.
- Explore the short-term prognostic implications of identifying a distributive-shock profile at presentation.

## 2. Methods

### 2.1. Study Design

This prospective observational pilot study was conducted at the Intermediate Medical Care Unit (IMCU) of Ospedale Alto Vicentino, Santorso, Italy, from 1 September 2024, through May 2025.

### 2.2. Patients

All consecutive patients with a diagnosis of community-acquired sepsis admitted from the Emergency Department (ED) to the IMCU were screened for inclusion. Sepsis was defined, in accordance with the latest Surviving Sepsis Campaign guidelines, as suspected or confirmed infection associated with a SOFA score ≥ 2 [3]. We enrolled adults with community-acquired sepsis and established the diagnosis on arrival to the IMCU. Following the latest guidelines, sepsis was conceptualized as life-threatening organ dysfunction due to infection, operationalized as an acute increase in the total SOFA score by ≥2 points [3]. Although originally derived in ICU cohorts, SOFA is widely used across hospital settings, including intermediate care units [3,17].

Exclusion criteria. Patients were excluded if any of the following applied:

(1) age < 18 years; (2) absence of informed consent; (3) suspected or confirmed pregnancy; (4) admission from locations other than the ED; (5) sepsis secondary to surgery or trauma within the previous 3 months; (6) ED length of stay > 6 h before IMCU admission; (7) administration of > 1,000 mL of intravenous fluids within the 3 h preceding IMCU admission; (8) active bleeding; (9) initiation of inotropic or vasopressor support prior to IMCU entry; (10) initiation of noninvasive ventilation prior to IMCU admission; (11) end-stage disease with life expectancy < 3 months; (12) a clinical indication for immediate organ support (e.g., endotracheal intubation) that was not instituted because of pre-existing chronic or palliative care decisions precluding transfer to the intensive care unit.

### 2.3. Study Protocol

#### 2.3.1. General Assessment

At enrollment, after obtaining written informed consent, demographic characteristics, medical history, and presenting clinical data were recorded in a dedicated electronic case report form. Routine blood tests performed in the ED for patients with suspected infection already included all variables required to compute the SOFA score, which was calculated and recorded as the baseline reference. Sepsis was defined as suspected or confirmed infection plus an increase in SOFA ≥2 points, consistent with the guideline’s definitions [3]. We did not use qSOFA for case definition.

Age, sex, body mass index (BMI), and major comorbidities (arterial hypertension, ischemic heart disease, peripheral artery disease, prior stroke, chronic heart failure, diabetes mellitus, chronic kidney disease, malignancy, and ongoing antithrombotic therapy) were documented and used to compute the Charlson Comorbidity Index (CCI). Vital signs at admission were captured both as individual values and aggregated into the National Early Warning Score (NEWS).

Concomitantly, an arterial line was placed, and an arterial blood sample was obtained for lactate measurement and blood gas analysis, including pH, partial pressure of oxygen (PaO_2_), partial pressure of carbon dioxide (PaCO_2_), bicarbonate (HCO_3_), and oxygen saturation (SaO_2_). In parallel, a venous sample was collected for inflammatory markers, C-reactive protein (CRP), white blood cell count (WBC), and procalcitonin (PCT); coagulation parameters (platelet count, PT-INR, aPTT, fibrinogen, D-dimer), complete blood count (hemoglobin [Hb], hematocrit [Hct]), and indices of renal and hepatic function (bilirubin, albumin, transaminases).

Based on the collected data, both the Acute Physiology and Chronic Health Evaluation II (APACHE II) and the SOFA scores were calculated at enrollment.

#### 2.3.2. Noninvasive Hemodynamic Assessment

Concurrently with clinical and laboratory assessment, hemodynamic status was measured noninvasively using the Noninvasive Cardiac System (NICaS®, NI-MEDICAL Ltd., Ra’anana, Israel; software version 3.63.15). The NICaS® device uses impedance cardiography (bioimpedance) to estimate beat-to-beat stroke volume from cyclic changes in transthoracic/whole-body electrical impedance produced by pulsatile blood flow; cardiac output/index and total peripheral resistance/index are then derived. Recordings were obtained on arrival at the IMCU with the patient supine and sensors applied to the volar aspect of both wrists. Skin was cleaned with swabs and hair removed if needed. Electrodes were placed according to the manufacturer’s wrist-to-wrist configuration on the volar aspect proximal to the carpal crease, with gentle pressure for ≥10 s to optimize contact. Signal acceptability required a stable impedance baseline and a device “good quality” indicator during a ≥60 s acquisition without visible motion artifacts; otherwise, measurements were repeated for up to 10 min. 

Acquired parameters included Stroke Index (SI), Cardiac Index (CI), Total Peripheral Resistance Index (TPRI), and Cardiac Power Index (CPI). When classifying values as reduced or increased, we used the adult reference intervals provided by the NICaS manufacturer and prior validation literature. In addition to absolute values, the device provided body size–indexed measures, allowing personalized assessment and comparison across patients with different body habitus. The reliability of NICaS measurements has been previously demonstrated against invasive thermodilution techniques [18,19,20,21]. If signal quality was inadequate, measurements were repeated for up to 10 min; if no reliable trace was obtained within this interval, the patient was excluded.

#### 2.3.3. Definition of the Distributive-Shock Hemodynamic Profile

Based on noninvasive indices at enrollment, a “distributive-shock hemodynamic profile” was defined by the coexistence of cardiac output (CO) > 8 L/min and TPRI < 770 dyn·s/cm^5^, as described in prior literature [22,23]. This combination was interpreted as indicating marked systemic vasodilation in the presence of high cardiac output, consistent with distributive shock.

#### 2.3.4. Assessment of Fluid Responsiveness

Following the initial noninvasive assessment, all patients received a weight-normalized test bolus of ~7 mL/kg (≈500 mL in a 70 kg adult) of isotonic crystalloid infused over 20 min, selected a priori to approximate the commonly used 500 mL fluid challenge and to enable standardized, time-bound hemodynamic reassessment; recent studies support the use of small, standardized boluses with reassessment in early sepsis resuscitation [24,25]. A second NICaS^®^ assessment was then performed. Fluid responsiveness was defined as a ≥15% increase in stroke volume (SV) from baseline; patients meeting this criterion were classified as fluid responders [24,25].

## 3. Outcomes

The first primary endpoint was the correlation between noninvasive hemodynamic parameters recorded at enrollment and macro-hemodynamic indices, namely the SOFA score and MAP.

The second endpoint was the determination of fluid responsiveness.

The third endpoint was 30-day mortality from enrollment, ascertained by direct contact with the patient or, when not feasible, with next of kin.

## 4. Ethical Considerations

The study was conducted in accordance with the Declaration of Helsinki and applicable institutional requirements. Written informed consent was obtained from all participants (or their legal representatives). Ethical approval was granted by the local ethics committee (Comitato Etico Territoriale dell’Area Sud Ovest Veneto, Italy) under protocol number 5017.

## 5. Statistical Analysis

Given the exploratory nature of the study, no formal sample-size calculation was performed; the cohort comprised all consecutive septic patients admitted to the IMCU who met eligibility criteria. This pilot study used consecutive enrollment within a predefined time window in a highly selected IMCU population (community-acquired sepsis admitted directly from the ED, with minimal pre-IMCU hemodynamic therapy per exclusion criteria), making an a priori size estimate imprecise and potentially misleading. Analyses were therefore planned as descriptive and univariable. Given the limited number of outcome events and fluid non-responders, multivariable modeling was not performed to avoid overfitting and unstable estimates (events-per-variable below accepted thresholds). Categorical variables were summarized as counts and percentages, and continuous variables as mean ± standard deviation (SD) or median with interquartile range (IQR), according to distribution.

Univariable comparisons used one-way ANOVA or the Kruskal–Wallis and Mann–Whitney U tests, as appropriate. Bonferroni correction was applied for post hoc multiple comparisons. Within-group variability was described using the coefficient of variation (CV%), and for nonparametric variables by a median-absolute-deviation-based coefficient of variation (MAD%).

To visualize the distribution of hemodynamic indices in septic patients, we employed violin plots and radar charts. For radar-plot visualization, variables with different units (SV, SI, CO, CI, CPI, TPR, TPRI) were robustly scaled to [0–1] using the cohort 5th–95th percentile range: s = (x − p5)/(p95 − p5); values < 0 and > 1 were truncated to 0 and 1, respectively. For interpretability, TPR and TPRI were reverse-coded (1 − s) so that larger radii indicate greater vasodilation/lower resistance. Lines depict group medians by SOFA tertiles.

We prespecified SOFA and MAP as the only comparators for correlation analyses. Other surrogates of perfusion (e.g., lactate) were not analyzed comparatively to avoid multiplicity.

Correlations between hemodynamic and macro-hemodynamic indices (SOFA and MAP) were explored using Pearson’s correlation for normally distributed variables and Spearman’s rank correlation otherwise. Correlation strength was categorized as weak (r < 0.30), moderate (0.30–0.50), or strong (r > 0.50).

Survival was analyzed using the Kaplan–Meier method and compared with the log-rank test. All statistical tests were two-sided, with *p* < 0.05 considered significant. All analyses were performed using STATA 16.1 (StataCorp LLC, College Station, TX, USA). Violin plots were generated in STATA using kernel-density estimates mirrored to form the violin; the same bandwidth and axis scaling were applied across groups/panels, and summary overlays indicate median and interquartile range.

All variables were complete; analyses were therefore conducted as complete-case with no imputation.

## 6. Results

A total of 115 patients were enrolled. Baseline characteristics are summarized in Supplementary Table S1. The cohort had a mean age of 70.9 ± 11.5 years and was predominantly male (63.5%). Mean body mass index was 21.5 ± 1.8 kg/m^2^, and the mean Charlson Comorbidity Index was 5.4 ± 2.8. At admission, the mean NEWS was 6.2 ± 3.1 and the mean MAP was 85.6 ± 16.7 mmHg; 15.7% (18/115) presented with MAP < 65 mmHg. Mean SOFA and APACHE II scores were 4.5 ± 2.2 and 12.9 ± 4.9, respectively. Median lactate was 1.87 mmol/L (IQR 1.40–2.62).

### 6.1. Hemodynamic Characteristics at Admission

At IMCU entry, mean stroke volume (SV) was 77.5 ± 21.5 mL, with 23.5% (27/115) below the reference range. SI had a median of 40.2 mL/m^2^ (IQR 31.9–47.1) and was low in 31.3% (36/115).

Median CO and CI were 6.8 L/min (IQR 5.8–8.3) and 3.6 L/min/m^2^ (IQR 3.1–4.3), respectively. Elevated CO was observed in 31.3% (36/115) and low CO in 2.6% (3/115). For CI, 35.7% (41/115) were above and 11.3% (13/115) below the reference range.

Mean total peripheral resistance (TPR) was 1,017.2 ± 367.8 dyn·s/cm^5^, and mean TPRI was 1,935.7 ± 741.7 dyn·s/cm^5^/m^2^. Reduced TPR was present in 26.1% (30/115) and increased TPR in 12.2% (14/115). Similarly, reduced TPRI occurred in 34.8% (40/115) and increased TPRI in 7.0% (8/115).

### 6.2. Association with SOFA at Admission

Patients were stratified into SOFA tertiles at entry, identifying three categories of increasing severity. The distribution of hemodynamic indices across tertiles is shown in Table 1.

Mean SV was similar across tertiles (79.8, 78.6, 71.9 mL; *p* = 0.287), with progressively higher dispersion (CV 27.7%, 25.2%, 32.1%). SI also did not differ significantly (41.7 vs. 39.9 vs. 37.5 mL/m^2^; *p* = 0.469), with the highest variability in the upper tertile (MAD–CV 19.9%). CO medians were overlapping (6.8, 7.1, 6.8 L/min; *p* = 0.955), as were CI medians (3.6, 3.5, 3.8 L/min/m^2^; *p* = 0.650). In contrast, peripheral resistance decreased with higher SOFA: TPR (1098.8, 993.1, 926.1 dyn·s/cm^5^; *p* = 0.131) and especially TPRI (2133.2, 1881.9, 1708.2 dyn·s·cm^−5^·m^−2^; *p* = 0.049), with a marked rise in the proportion with reduced TPRI (from 15.9% to 57.1%; *p* = 0.001). Dispersion for resistance indices was substantial (CV up to 45.8%). CPI medians were comparable (0.71, 0.66, 0.62 W/m^2^; *p* = 0.220), with heterogeneous variability (CV 16.8–25.8%).

**Figure 1:**
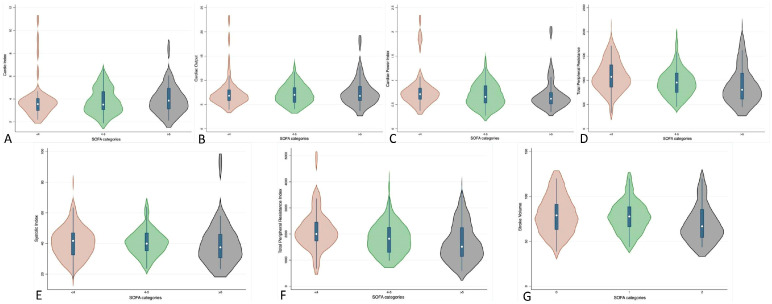
Violin plots of noninvasive hemodynamic indices at IMCU admission across SOFA tertiles (SOFA < 4, SOFA 4–5, SOFA > 5). (**A**) Heart rate; (**B**) Mean arterial pressure; (**C**) Stroke volume index; (**D**) Cardiac index; (**E**) Systemic vascular resistance index; (**F**) dP/dt (contractility index); (**G**) Cardiac power index.

Violin-plot visualizations (Figure 1) further demonstrate the within-tertile heterogeneity: each parameter (SV, SI, CO, CI, CPI, TPR, TPRI) shows wide, largely overlapping distributions across SOFA categories, and aside from the trend toward lower TPR/TPRI, no clear monotonic progression emerges. 

Each panel shows the distribution of SV, SI, CO, CI, CPI, TPR, and TPRI in their original units; wider sections indicate higher data density and the central line marks the median. Distributions are broad and largely overlapping within and across tertiles; aside from a shift toward lower TPR/TPRI with increasing SOFA, no consistent monotonic gradient is evident. Numerical medians and interquartile ranges for each variable are reported in Table 1.

**Figure 2:**
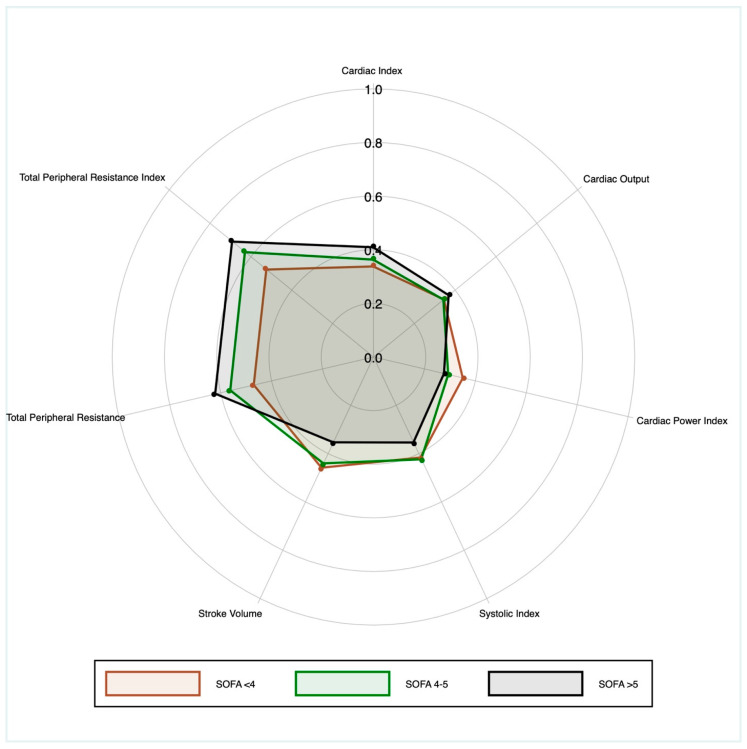
Radar plot of noninvasive hemodynamic indices at IMCU admission by SOFA tertiles. Each variable was normalized to the cohort p5–p95 range and expressed on a unitless 0–1 scale for visualization; values outside this interval were truncated. TPR and TPRI were reverse-coded (1 − s) so that larger radii reflect greater vasodilation/lower resistance. Lines show medians for each SOFA tertile. Concentric rings are fixed reference levels used uniformly across parameters to facilitate visual quantification; values are plotted on the same scale for all axes.

Patients with the same SOFA score exhibited markedly different hemodynamic phenotypes, with broad and frequently overlapping distributions across tertiles. No clear monotonic gradient was evident from the lowest to the highest tertile. Figure 2 further illustrates the substantial within-SOFA heterogeneity of hemodynamic profiles.

Correlation analyses between SOFA at enrollment and noninvasive hemodynamic indices showed absent to weak associations (Table 2).

SV and SI were not significantly correlated with SOFA (*p* = 0.141 and 0.310). CO and CI likewise showed no association (*p* = 0.731 and 0.351). TPR and TPRI exhibited weak inverse correlations with SOFA (r = −0.198, *p* = 0.034; r = −0.241, *p* = 0.009). CPI trended negatively but did not reach significance (*p* = 0.085).

With MAP, there were no significant correlations for SV, SI, CO, or CI (all *p* > 0.20). In contrast, TPR and TPRI correlated positively and significantly with MAP (r = 0.461 and 0.547, both *p* < 0.001). CPI also showed a strong positive association with MAP (ρ = 0.550, *p* < 0.001).

### 6.3. Distributive-Shock Hemodynamic Profile at Admission

A distributive-shock hemodynamic profile was present in 21.7% (25/115) of patients. Mean SOFA was 4.2 ± 2.1 in those without the profile versus 5.4 ± 2.3 in those with it (*p* = 0.016). Prevalence increased across SOFA categories: 9.1% (4/44) for SOFA < 4, 27.9% (12/43) for SOFA 4–5, and 32.1% (9/28) for SOFA > 5 (*p* = 0.033).

Mean MAP was 82.4 ± 15.5 mmHg in patients with the profile versus 86.4 ± 16.9 mmHg in those without (*p* = 0.289). Notably, among patients with a distributive-shock profile, only 20% (5/25) had MAP < 65 mmHg at admission (*p* = 0.538).

### 6.4. Association with Fluid-Challenge Response

Fluid non-responders accounted for 27.8% (32/115). The distribution of hemodynamic indices by fluid responders vs. non-responders is reported in Table 3.

Mean SV was similar between groups (78.4 vs. 74.9 mL; *p* = 0.429), with comparable rates of reduced SV (22.9% vs. 25.0%; *p* = 0.810). Median SI was 40.2 vs. 38.7 mL/m^2^ (*p* = 0.820), with no difference in the prevalence of reduced SI. Median CO was slightly higher in non-responders (7.5 vs. 6.6 L/min; *p* = 0.232), whereas CI was significantly higher in non-responders (4.1 vs. 3.4 L/min/m^2^; *p* = 0.015), and the proportion with increased CI was also greater (56.3% vs. 27.7%; *p* = 0.004). TPR and the TPRI were lower in non-responders, with significant between-group differences (TPR *p* = 0.050; TPRI *p* = 0.019) and a higher frequency of reduced values (reduced TPR 46.9% vs. 18.1%; *p* = 0.002; reduced TPRI 62.5% vs. 24.1%; *p* < 0.001). CPI did not differ between groups. Finally, a distributive-shock hemodynamic profile at admission was present in 15.7% (13/83) of fluid responders versus 37.5% (12/32) of non-responders (*p* = 0.011).

### 6.5. Hemodynamic Indices, Distributive-Shock Profile, and 30-Day Mortality

Thirty-day mortality was 14.8% (17/115). Baseline characteristics by survival status are summarized in Table 4.

Non-survivors had a lower SV at admission (67.3 vs. 79.2 mL; *p* = 0.034). No statistically significant differences were observed for CO, CI, or CPI. Mean TPR and TPRI were similar between groups, although point estimates for TPRI trended lower in non-survivors.

**Figure 3:**
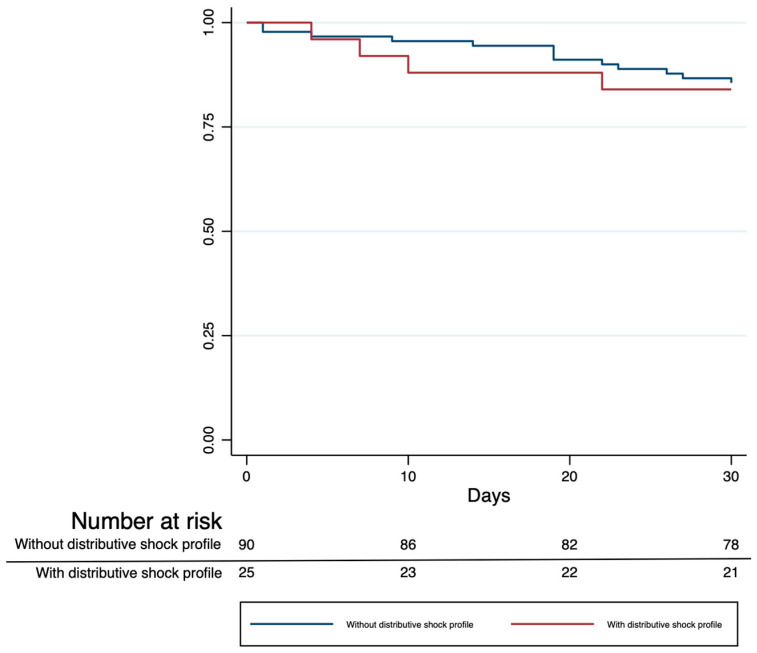
Kaplan–Meier curves for 30-day survival stratified by the presence of a distributive-shock hemodynamic profile at admission. Numbers at risk are reported below the x-axis. Censoring is indicated by tick marks.

A distributive-shock hemodynamic profile at admission was present in 21.4% (21/98) of 30-day survivors and 23.5% (4/17) of non-survivors (*p* = 0.846). Kaplan–Meier analysis showed no significant difference in survival between patients with and without the distributive-shock profile (log-rank *p* = 0.808; Figure 3).

## 7. Discussion

In this exploratory observational study of patients with community-acquired sepsis, the initial hemodynamic profile did not correlate with macro-hemodynamic indices currently used to estimate perfusion deficits and related organ dysfunction. Our findings suggest that patients uniformly classified as septic may in fact display wide differences in hemodynamic phenotype and, by extension, in perfusion status. If confirmed, these results would have relevant clinical implications, pointing toward personalization of hemodynamic support, which is presently applied rather uniformly across septic patients without accounting for individual physiology.

The cohort comprised non-surgical, non-traumatic community sepsis patients admitted directly from the ED to the IMCU before substantial fluid loading, vasoactive support, or ventilatory assistance. This design allowed us to examine the intrinsic hemodynamics of sepsis at presentation, at a time when the pathophysiological picture is less likely to be distorted by treatments or overlapping conditions [26]. Unlike trials centered on critically ill patients in the ICU, from which many current recommendations derive, the present population reflects the sepsis commonly managed in general medical wards [26,27]. These patients are often under-represented in clinical research yet constitute the majority of hospitalized sepsis cases [28]. Without timely, appropriate management, they may evolve toward organ failure or hemodynamic instability and later contribute to the populations enrolled in high-intensity settings. Tools currently in widespread use, SOFA score and MAP, largely extrapolated from intensive-care contexts, may therefore be less effective at capturing early hemodynamic complexity, as also suggested by the broad mortality gradients observed within the same SOFA category. Unlike most ICU-based studies, these data come from an intermediate care setting, which reflects where the majority of sepsis patients are initially managed. In this scenario, early, noninvasive hemodynamic assessment can provide a higher-resolution perspective to guide tailored interventions from the first hours [29].

This study offers several insights.

Firstly, septic patients exhibit markedly heterogeneous hemodynamic profiles. Despite sharing a uniform diagnostic label, individuals may present with profoundly different perfusion states. Within a single nosological category that, according to current recommendations, triggers a homogeneous therapeutic pathway lie pathophysiological nuances that are not captured by conventional macro-hemodynamic indices or scores yet may matter clinically. In a prospective study of 100 patients with acute circulatory failure (55 septic), hypovolemia was found in 60% of septic vs. 51% of non-septic patients, indicating preload deficit as a frequent, but not exclusive, pattern [30]. A hyperkinetic vasoplegic profile was present in only 31%, and early left-ventricular dysfunction in 30% [30]. Even with preserved or increased ejection fraction, stroke volume may be reduced because of inadequate preload due to increased vascular permeability and excessive interstitial filtration [31,32]. In pediatric sepsis, Rao et al. reported vasodilatory shock (CI > 5.5 L·min^−1^·m^−2^, SVR*I < 1000 dyn·s·cm^−5^·m^−2^) in 53.3% and vasoconstrictive shock (CI < 3.3 and SVR*I > 1600) in 46.7% [33]. Similarly, Geri et al. identified five hemodynamic clusters in 360 patients with septic shock, 23.3% hyperkinetic, 22.5% right-ventricular dysfunction, 19.4% persistently hypovolemic, 17.7% left-systolic dysfunction (ICU mortality 50%), and only 16.9% well-resuscitated [34]. Collectively, these data support the view that sepsis encompasses distinct, dynamic circulatory phenotypes with management relevance.

Secondly SOFA and MAP showed no meaningful correlations with noninvasively derived hemodynamic indices in our cohort. Our findings demonstrate within-SOFA hemodynamic heterogeneity: patients with identical SOFA values may have markedly different noninvasive cardiovascular profiles. This is coherent with Surviving Sepsis Campaign guidance, which frames SOFA as an organ-dysfunction construct rather than a direct perfusion metric, and underlines the potential value of noninvasive hemodynamic monitoring to tailor resuscitation strategies. The presence of a distributive-shock profile, operationalized by high CO and low TPRI, was neither predictable from, nor systematically associated with, alterations in these macro-indices. While SOFA and MAP are widely used for risk stratification and, at times, to inform treatment, prior studies have highlighted their limited physio-pathological specificity, particularly in describing the underlying perfusion state. Ferreira et al. observed a linear association between rising SOFA and mortality but also substantial outcome dispersion within each SOFA value [35]. Kaukonen et al. reported that roughly one-third of patients with SOFA ≥ 2 had surprisingly low mortality, questioning the score’s sensitivity as a stand-alone identifier of severe illness [36]. By aggregating physiologically heterogeneous patients, SOFA can miss key facets such as microcirculatory dysfunction or vascular tone, and the inclusion of such dimensions has been shown to improve predictive performance [37,38]. Nouriel et al. found only moderate correlation between blood-pressure variability and SOFA (r = 0.544; *p* = 0.024) in septic patients, underscoring that static severity scores describe organ failure but not its hemodynamic underpinnings [39]. In pediatrics, Chandran et al. showed that electrical cardiometry discriminated septic from hypovolemic shock (lower CO and SI, altered HR variability) despite comparable MAP between groups [39]. Together with our data, these observations indicate that MAP and SOFA, while clinically useful, do not discriminate hemodynamic phenotypes, supporting the need for early integration with more sensitive, physiology-focused monitoring.

Thirdly, hemodynamic indices measured at presentation, and the mere presence of a distributive-shock profile at arrival, were not associated with 30-day mortality. 

This is not unexpected: classic work by Parker et al. showed that both survivors and non-survivors in early severe sepsis had elevated cardiac index, reduced systemic vascular resistance, and stroke volume index within normal ranges, with no substantial outcome differences in the early phase [40]. More recent studies likewise report that the initial hemodynamic profile in the critically ill is not a reliable predictor of short-term mortality [41,42,43]. These indices are highly dynamic, rapidly modulated by the clinical course and by therapies often guided by the same measurements, which limits their longer-term prognostic value. In contrast, markers such as SOFA and MAP may reflect more chronic or advanced organ dysfunction, less amenable to rapid correction in severe disease. This perspective reinforces the idea that noninvasive hemodynamic assessment should be considered an operational tool to guide individualized, adaptive treatment, rather than a simple prognostic marker. While exploratory, these data support testing protocols that use early noninvasive hemodynamic profiling at IMCU admission to guide initial therapeutic choices, for example, balancing vasopressors versus additional fluids based on flow and vascular-tone patterns, within pragmatic, prospective studies. Beyond impedance cardiography, several bedside noninvasive modalities can complement early hemodynamic assessment and inform therapy. Focused echocardiography/Doppler allows rapid estimation of stroke volume/cardiac output and ventricular function, while multi-organ ultrasound integrates perfusion and congestion signs; dynamic tests (e.g., passive leg raise or a small fluid challenge) coupled with a real-time flow measurement outperform static parameters for assessing fluid responsiveness. Simple adjuncts such as capillary refill time and peripheral perfusion signs can further support decision-making when advanced monitoring is unavailable [3]. In parallel, early antimicrobials and initial fluid resuscitation, as recommended by the Surviving Sepsis Campaign, can rapidly reshape circulatory profiles (e.g., source control reducing vasoplegia; preload optimization increasing stroke volume in responders), so noninvasive indices should be interpreted in the context of treatment timing [3]. Our study captured hemodynamics at IMCU arrival but was not designed to model time-to-antibiotics or cumulative fluids; these exposures are potential effect modifiers and merit prospective capture in future studies.

For clinicians in IMCU settings, pragmatic messages emerge: (1) SOFA/MAP alone are insufficient surrogates of the underlying hemodynamics and should be complemented by early noninvasive profiling; (2) a distributive-consistent profile can be present despite MAP ≥ 65 mmHg, so treatment choices should consider flow and vascular-tone indices alongside timing of antibiotics and fluids. These hypotheses require confirmation in larger, protocolized studies.

## 8. Limitations

The single-center design may introduce biases related to local organization and care processes. Nevertheless, uniform selection criteria and a standardized clinical pathway likely mitigated these effects. This pilot study was not designed to establish definitive prognostic markers but to demonstrate feasibility, describe the spectrum of hemodynamic heterogeneity, and generate hypotheses for targeted management strategies. The macro-hemodynamic indices chosen for correlation analyses were restricted to SOFA and MAP, excluding other proposed markers of perfusion impairment (e.g., lactate, albumin). Our analyses intentionally excluded direct comparisons with microcirculatory/metabolic surrogates such as lactate and capillary refill time, to keep the study focused on macro-hemodynamic profiling and because these surrogates were not captured under a standardized research protocol in our IMCU. Future work integrating standardized capillary refill time/lactate alongside noninvasive hemodynamic indices is warranted. Focused echocardiography was not performed and recorded according to a standardized protocol across all patients; when obtained, examinations were performed at clinicians’ discretion and were not captured in a structured case-report form. Future studies will incorporate a standardized echocardiography workflow to integrate structural and functional cardiac data with noninvasive hemodynamic indices. NICaS measurements, while previously validated, may be influenced by peripheral vasoplegia or by technical factors such as electrode contact; replication with alternative noninvasive techniques would strengthen generalizability. We used a pre-specified ~7 mL/kg crystalloid bolus (≈500 mL for a 70 kg adult) for standardization and did not compare alternative bolus sizes or infusion rates, which may influence short-term hemodynamic responses. By excluding patients who had already received ≥1000 mL of fluids or any vasopressors prior to IMCU admission, we may have under-represented the most severe hemodynamic phenotypes and shifted the observed distribution of profiles; this selection was intentional to characterize early, relatively treatment-naïve physiology but may limit generalizability to more advanced shock. We prioritized SOFA and MAP because they are guideline-endorsed references for sepsis diagnosis and management and thus widely used not only for prognosis but also operationally. Finally, we did not account for minor procedures prior to enrollment (e.g., small-volume fluids, brief vasopressor exposure). However, stringent inclusion/exclusion criteria yielded a cohort largely naïve to hemodynamic treatment, reducing the impact of this potential bias.

## 9. Conclusions

In this cohort of patients with community-acquired sepsis, individuals who shared the same diagnostic label displayed markedly heterogeneous hemodynamic phenotypes at presentation. This heterogeneity exposes a limitation of current guideline-driven strategies, which often advocate uniform resuscitation pathways despite potentially divergent perfusion states. Macro-indices with recognized prognostic value, SOFA and MAP, showed poor correlation with noninvasive hemodynamic measures, indicating that clinically similar categories can conceal distinct circulatory profiles.

Taken together, these findings support viewing hemodynamics as a dynamic, modifiable dimension to be measured early and used operationally to individualize fluid and vasoactive therapy, rather than as a static predictor of short-term mortality. Prospective interventional studies are warranted to test whether early noninvasive hemodynamic profiling, including indices such as the TPRI, can improve patient-centered outcomes by guiding personalized management in sepsis. These findings underscore the potential of early noninvasive hemodynamic assessment to detect perfusion phenotypes not captured by standard scores. Future interventional studies are warranted to determine whether such phenotyping can improve patient outcomes.

## Figures and Tables

**Table 1:** Hemodynamic indices at IMCU admission by SOFA tertiles.

Index	SOFA < 4	SOFA 4–5	SOFA > 5	*p*-Value
Patients, n (%)	44 (38.3)	43 (37.4)	28 (24.3)	
SV, Mean (SD), mL	79.8 (22.1)	78.6 (19.8)	71.9 (23.1)	0.287
CV%	27.7	25.2	32.1	
SV reduced, n (%)	8 (18.2)	8 (18.6)	11 (39.3)	0.081
SI, Median (IQR), mL/m^2^	41.7 (32.1–47.1)	39.9 (35.1–47.1)	37.5 (30.6–46.4)	0.469
CV (MAD%)	17.8	13.5	19.9	
SI reduced, n (%)	15 (34.1)	10 (23.3)	11 (39.3)	0.338
SI increased, n (%)	1 (2.3)	2 (4.7)	1 (3.6)	0.835
CO, Median (IQR), L/min	6.8 (5.8–8.1)	7.1 (5.4–8.5)	6.8 (5.8–8.8)	0.955
CV%	16.6	21.7	17.8	
CO reduced, n (%)	1 (2.3)	0 (0)	2 (7.1)	0.250
CO increased, n (%)	13 (29.5)	14 (32.6)	9 (32.1)	0.967
CI, Median (IQR), L/min/m^2^	3.6 (2.9–4.1)	3.5 (3.1–4.7)	3.8 (3.1–5.1)	0.650
CV%	14.2	23.7	20.9	
CI reduced, n (%)	5 (11.4)	5 (11.6)	3 (10.7)	1.000
CI increased, n (%)	12 (27.3)	17 (39.5)	12 (42.9)	0.297
TPR, Mean (SD), dyn × s/cm^5^	1098.8 (353.2)	993.1 (339.3)	926.1 (416.1)	0.131
CV%	32.1	34.2	44.9	
Reduced TPR, n (%)	4 (9.1)	13 (30.2)	13 (46.4)	0.001
Increased TPR, n (%)	6 (13.6)	4 (9.3)	4 (14.3)	0.816
TPRI, Mean (SD), dyn × s × cm^−5^ × m^−2^	2133.2 (751.1)	1881.9 (666.6)	1708.2 (781.9)	**0.049**
CV%	35.2	35.4	45.8	
Reduced TPRI, n (%)	7 (15.9)	17 (39.5)	16 (57.1)	**0.001**
Increased TPRI, n (%)	5 (11.4)	2 (4.7)	1 (3.6)	0.455
CPI, Median (IQR), W/m^2^	0.71 (0.61–0.85)	0.66 (0.53–0.89)	0.62 (0.51–0.77)	0.220
CV%	16.8	25.8	17.7	
CPI reduced, n (%)	3 (6.8)	7 (16.3)	2 (7.1)	0.343
CPI increased, n (%)	6 (13.6)	6 (14)	4 (14.3)	1.000

Bold *p*-values denote statistically significant results at the 0.05 level.

**Table 2:** Correlations of hemodynamic indices with SOFA and MAP at admission.

Index	Correlation with SOFA	*p*-Value	Correlation with MAP	*p*-Value
SV, Pearson	−0.138	0.141	0.070	0.455
SI, Spearman	−0.096	0.310	−0.064	0.496
CO, Spearman	0.032	0.731	0.110	0.244
CI, Spearman	0.088	0.351	−0.003	0.974
TPR, Pearson	−0.198	0.034	0.461	**<0.001**
TPRI, Pearson	−0.241	0.009	0.547	**<0.001**
CPI, Spearman	−0.161	0.085	0.550	**<0.001**

Bold *p*-values denote statistically significant results at the 0.05 level.

**Table 3:** Hemodynamic indices at admission by response to the fluid challenge.

Index	Fluid Responder	Fluid Non-Responder	*p*-Value
Patients, n (%)	83 (72.2)	32 (27.8)	
SV, mean (SD) mL	78.4 (20.9)	74.9 (23.2)	0.429
SV reduced, n (%)	19 (22.9)	8 (25)	0.810
SI, median (IQR), mL/m^2^	40.2 (31.4–47)	38.7 (33.1–47)	0.820
SI reduced, n (%)	26 (31.3)	10 (31.3)	1.000
SI increased, n (%)	1 (1.2)	4 (9.4)	0.065
CO, median (IQR), L/min	6.6 (5.7–8.1)	7.5 (5.9–8.8)	0.232
CO reduced, n (%)	3 (3.6)	0 (0.0)	0.276
CO increased, n (%)	23 (27.7)	13 (40.6)	0.262
CI, median (IQR), L/min/m^2^	3.4 (3.1–4.2)	4.1 (3.6–5.3)	**0.015**
CI reduced, n (%)	9 (10.8)	4 (12.5)	1.000
CI increased, n (%)	23 (27.7)	18 (56.3)	**0.004**
TPR, mean (SD), dyn × s/cm^5^	1057.3 (332.1)	913.1 (436.2)	0.050
TPR reduced, n (%)	15 (18.1)	15 (46.9)	**0.002**
TPR increased, n (%)	11 (13.3)	3 (9.4)	0.569
TPRI, mean (SD), dyn × s × cm^−5^×m^−2^	2035.7 (640.8)	1676.3 (916.8)	**0.019**
TPRI reduced, n (%)	20 (24.1)	20 (62.5)	**<0.001**
TPRI increased, n (%)	6 (7.2)	2 (6.3)	0.853
CPI, median (IQR), W/m^2^	0.68 (0.53–0.84)	0.70 (0.52–0.89)	0.673
CPI reduced, n (%)	8 (9.6)	4 (12.5)	0.375
CPI increased, n (%)	10 (12)	6 (18.8)	0.352

Bold *p*-values denote statistically significant results at the 0.05 level.

**Table 4:** Hemodynamic indices at IMCU admission by 30-day survival.

Index	Survivors at 30 Days	Non-Survivors at 30 days	*p*-Value
Patients, n (%)	98 (85.2)	17 (14.8)	
SV, mean (SD) mL	79.2 (21.1)	67.3 (22.1)	**0.034**
SV reduced, n (%)	20 (20.4)	7 (41.2)	0.116
SI, median (IQR), mL/m^2^	40.8 (33.1–47.1)	37.1 (29.6–41.8)	0.112
SI reduced, n (%)	29 (29.6)	7 (41.2)	0.399
SI increased, n (%)	3 (3.1)	1 (5.9)	0.477
CO, median (IQR), L/min	6.9 (5.8–8.5)	6.7 (4.8–8.1)	0.289
CO reduced, n (%)	1 (1.1)	2 (11.8)	0.057
CO increased, n (%)	32 (32.7)	4 (23.5)	0.577
CI, median (IQR), L/min/m^2^	3.6 (3.1–4.3)	3.5 (2.8–4.6)	0.915
CI reduced, n (%)	9 (9.2)	4 (23.5)	0.101
CI increased, n (%)	35 (35.7)	6 (35.3)	1.000
TPR, mean (SD), dyn × s/cm^5^	1016.6 (355.9)	1020.6 (442.4)	0.967
TPR reduced, n (%)	15 (18.1)	15 (46.9)	**0.002**
TPR increased, n (%)	11 (13.3)	3 (9.4)	0.569
TPRI, mean (SD), dyn × s × cm^−5^×m^−2^	1960.8 (732.9)	1790.8 (797.8)	0.385
TPRI reduced, n (%)	25 (25.5)	5 (29.4)	0.768
TPRI increased, n (%)	10 (10.2)	4 (23.5)	0.219
CPI, median (IQR), W/m^2^	0.71 (0.55–0.85)	0.54 (0.47–0.83)	0.077
CPI reduced, n (%)	9 (9.2)	3 (17.6)	0.382
CPI increased, n (%)	13 (13.3)	3 (17.6)	0.706

Bold *p*-values denote statistically significant results at the 0.05 level.

## Data Availability

Data are available on request due to privacy/ethical restrictions.

## Supplementary Materials

The following supporting information can be downloaded at: https://www.mdpi.com/article/10.3390/healthcare13212686/s1, Table S1. Baseline characteristics of the study population.
