# Supplementary material for: Hemodynamic Heterogeneity in Community-Acquired Sepsis at Intermediate Care Admission: A Prospective Pilot Study Using Impedance Cardiography
Source: Healthcare (Basel). 2025 Oct 23;13(21):2686. doi: 10.3390/healthcare13212686 (PMC12610071; doi:10.3390/healthcare13212686)
Supplement: Supplementary file 1 [file healthcare-13-02686-s001.zip › healthcare-3910182-supplementary.pdf]

Table S1. Baseline characteristics of the study population.

**Variable**	
Patients, n (%)	115 (100)
Age, years, mean (SD)	70.9 (11.5)
Sex, n (%)	
Male	73 (63.5)
Female	42 (36.5)
Anthropometrics	
Weight, kg, mean (SD)	79.2 (20.5)
Height, m, mean (SD)	1.71 (0.09)
BMI, kg/m^2, mean (SD)	21.5 (1.8)
Charlson Comorbility Index, points, mean (SD)	5.4 (2.8)
Vital signs	
Systolic BP, mmHg, mean (SD)	114.7 (23.5)
Diastolic BP, mmHg, mean (SD)	68.4 (14.4)
Heart rate, bpM, mean (SD)	93.4 (14.4)
Peripherial O_2 saturation, %, mean (SD)	94.6 (3.7)
Respiratory rate, breaths/min, median (IQR)	20 (18-25)
MAP, mmHg, mean (SD)	85.6 (16.7)
NEWS, point, mean (SD)	6.2 (3.1)
SOFA, point, mean (SD)	4.5 (2.2)
Lactate, median (IQR)	1.87 (1.4-2.62)
APACHE II, point, mean (SD)	12.9 (4.9)
